# Long COVID prevalence among U.S. adults: A state-level ecological analysis of associations with SARS-CoV-2 incidence, COVID-19 hospitalization rates, COVID-19 vaccine coverage, and multimorbidity

**DOI:** 10.1371/journal.pone.0345433

**Published:** 2026-11-03

**Authors:** Xinmeng Zhao, Li Deng, Nicole D. Ford, Sharon Saydah

**Affiliations:** Coronavirus and Other Respiratory Viruses Division, National Center for Immunization and Respiratory Diseases, Centers for Disease Control and Prevention, Atlanta, Georgia, United States of America; National Center for Global Health and Medicine, JAPAN

## Abstract

**Background:**

Long COVID has emerged as a major public health concern in the United States, yet geographic variation in its prevalence remains poorly understood. This study examines how state-level differences in COVID-19 vaccination, SARS-CoV-2 infection burden, severity of acute COVID-19 (measured by hospitalization rates) and multimorbidity burden relate to adult Long COVID prevalence in the United States.

**Methods:**

We conducted an ecological analysis using data from the 2023 Behavioral Risk Factor Surveillance System (BRFSS), from which we estimated state-level prevalence of self-reported Long COVID among adults. These estimates were linked with publicly available data on SARS-CoV-2 incidence, COVID-19 hospitalizations, COVID-19 vaccine coverage, and a multimorbidity indicator (defined as ≥3 chronic conditions associated with increased risk of severe COVID-19, e.g., diabetes, obesity, chronic kidney disease). Multivariable linear regression models were used to assess the association between these factors and Long COVID prevalence, adjusting for state age and sex distributions. Rubin’s rules were applied to propagate the uncertainty in the estimates of Long COVID and COVID-19 vaccine coverage through regression analyses.

**Results:**

All examined factors—including SARS-CoV-2 incidence, COVID-19 hospitalization, COVID-19 vaccine coverage, and multimorbidity prevalence—varied by state. In univariable analyses, vaccine coverage and multimorbidity prevalence were significantly associated with Long COVID prevalence. In the multivariable model, vaccine coverage and SARS-CoV-2 incidence were significant; however, after accounting for uncertainty in Long COVID and vaccine coverage estimates, only higher vaccine coverage remained significantly associated with lower Long COVID prevalence.

**Conclusions:**

Higher State-level variation in COVID-19 vaccine coverage was consistently associated with lower adult Long COVID prevalence. These ecological findings support further investigation of vaccination and other state-level determinants of Long COVID burden and may inform population-level strategies for addressing Long COVID.

## Introduction

### Background

Long COVID, defined as new or persisting symptoms such as fatigue, headache, dyspnea and anosmia that last three or more months after acute SARS-CoV-2 infection [1], has emerged as a major public health concern in the United States. According to the 2023 Behavioral Risk Factor Surveillance System (BRFSS), age- and sex-standardized adult Long COVID prevalence ranged widely across the United States [2]. Similar levels of geographic heterogeneity have been documented across prior BRFSS cycles and other national surveys [3], highlighting consistent state-to-state variation in adult Long COVID prevalence.

Several factors have been reported to be associated with an increased likelihood of Long COVID, including SARS-CoV-2 infection, acute COVID-19 illness severity [4], vaccination status [4–6], and pre-existing chronic conditions—such as diabetes, heart disease, and obesity [4,7,8]. These factors may contribute to the observed geographic variation in Long COVID prevalence. While many studies have examined individual-level risk factors for Long COVID, few have attempted to quantify associations with Long COVID prevalence at the population level. It remains unclear whether geographic variation in Long COVID prevalence reflects underlying differences in these factors. Understanding these associations is critical for helping state and local health departments identify and support populations at higher risk and develop targeted prevention strategies.

Currently, there is no single integrated dataset that captures all relevant individual-level factors associated with Long COVID while providing coverage across all U.S. states [3]. Given this data gap, an ecological approach that leverages publicly available, aggregate data provides a policy-relevant alternative. By synthesizing state-level indicators of SARS-CoV-2 infection burden, acute COVID-19 illness severity (measured by hospitalization rates), COVID-19 vaccine coverage, and multimorbidity prevalence, it is possible to assess whether the observed geographic variation in Long COVID prevalence is associated with variation in these factors. This approach may provide valuable information for guiding public health interventions in the absence of comprehensive datasets with person-level data.

The objective of this study is to assess the association between state-level variation in adult Long COVID prevalence and state-level indicators of SARS-CoV-2 incidence, acute COVID-19 illness severity, COVID-19 vaccine coverage, and multimorbidity prevalence.

## Methods

### Study design and setting

We conducted an ecological study of adult Long COVID prevalence across U.S. states, using the state as the geographic unit. The primary aim was to identify factors associated with geographic variation in Long COVID burden using publicly available, population-level data sources.

### Outcome measure

The primary outcome was the crude, state-level prevalence of Long COVID among adults, based on data from 2023 BRFSS [9], with data collection extending into early 2024 (January through March, depending on the state). BRFSS is a state-based system of telephone health surveys that collects information on health risk behaviors, preventive health practices, and health care access. It is designed to produce representative estimates for non-institutionalized adults aged 18 years and older in participating states and territories.

Respondents were asked if they ever tested positive for COVID-19 or had been told by a doctor or other health care provider that they have or had COVID-19. Those who provided an affirmative response were then asked if they experienced symptoms lasting 3 months or longer that were not present prior to having COVID-19. If yes, respondents reported whether they were still experiencing symptoms at the time of interview (i.e., current Long COVID).

Final BRFSS sampling weights were applied to compute state-specific prevalence estimates and standard errors to make the sample statistics representative of the state or territory population from which it was drawn. The 2023 BRFSS public-use dataset excluded Kentucky and Pennsylvania due to insufficient data collection, resulting in a final analytic dataset comprising 48 states.

### Potential state-level factors associated with long COVID

To align with the 2023 BRFSS survey timing and the definition of Long COVID, we defined the analytic window as October 1, 2022, to September 30, 2023. This interval was selected to capture factors potentially associated with Long COVID preceding and overlapping with the timeframe relevant to the Long COVID definition, ensuring temporal alignment with prevalence measured in 2023. Final variables were selected based on data availability and represent the best feasible synthesis across the full analytic period and geographic scope. A summary of each data source and data processing is provided in S1 Text, Table A, with an overview presented below.

To quantify SARS-CoV-2 infection burden, we obtained state-level counts of new COVID-19 cases (confirmed and probable cases) from the Centers for Disease Control and Prevention (CDC) COVID-19 Response Data [10]. Mandatory COVID-19 reporting requirements ceased after May 10, 2023, following the official declaration of the end of the public health emergency. Iowa stopped reporting earlier, on April 1, 2023, and five additional states missed one week of reporting. To reduce the influence of incomplete reporting after the end of mandatory reporting, we aggregated reported incident cases from October 1, 2022, through May 10, 2023. SARS-CoV-2 incidence was calculated as cumulative reported cases per person-year using 2022 state population estimates [11].

To capture acute COVID-19 illness severity, we used state-specific weekly adult COVID-19 hospitalization rates per 100,000 persons computed based on data from the National Healthcare Safety Network (NHSN) [12]. NHSN is a national surveillance system that collects real-time hospitalization data from participating facilities across the United States. Hospitalization data were complete for all states and weeks included in the analysis. For comparability across measures with other factors, we calculated the cumulative hospitalization rate over the full analytic period (October 1, 2022, to September 30, 2023) and expressed this measure as COVID-19 hospitalizations per person-year.

For COVID-19 vaccine coverage, we used monthly, state-specific estimates from the National Immunization Survey (NIS) [13], a set of telephone-based surveys designed to monitor vaccination coverage in children, adolescents, and adults in the United States. Specifically, we used data from the NIS–Adult COVID Module (NIS-ACM). To improve the reliability of state-specific estimates of COVID-19 bivalent booster coverage by the end of the 2022–23 season, we used NIS-ACM data collected from March through June 2023 among adults aged ≥18 years. Point estimates and 95% confidence intervals were calculated using SUDAAN version 11.04, accounting for the complex survey design and survey weights to estimate receipt of a COVID-19 bivalent booster vaccine among adults during the 2022–23 season. Throughout this analysis, COVID-19 vaccine coverage refers to adult COVID-19 bivalent booster coverage during the 2022–23 season, estimated among all adults aged ≥18 years. This measure was selected to capture recent vaccination during the analytic period rather than historical vaccine coverage (i.e., ever vaccinated for COVID-19). Emerging evidence suggests that more recent COVID-19 vaccination is associated with a reduced risk of developing Long COVID [14]. The data used in the analysis are provided in S1 Text, Table B.

For chronic conditions, we used a composite comorbidity indicator representing the prevalence of adults with three or more chronic health conditions, hereafter referred to as “multimorbidity”, from unpublished 2024 estimates produced by the CDC’s Center for Forecasting and Outbreak Analytics (CFA). The estimates synthesized data from multiple data sources including 2022−2023 medical claims, consumer records, BRFSS, and the National Health Interview Survey (NHIS) to better estimate state-level comorbidity prevalence and covered a broader set of conditions relevant to severe COVID-19. The comorbidity conditions included asthma, obesity, diabetes, heart condition, current or former smoking, tuberculosis, cerebrovascular disease, chronic liver disease, chronic kidney disease, chronic lung disease, mental health disorder, cystic fibrosis, HIV, immunodeficiency, transplantation, immunosuppression, cancer, pregnancy, disability and neurocognitive or dementia-related conditions. The data used in this analysis are provided in S1 Text, Table C.

Although BRFSS allows for age- and sex-standardized estimation of Long COVID prevalence, and similar standardization was possible for multimorbidity, comparable data were unavailable for other state-level variables, including SARS-CoV-2 case incidence and COVID-19 hospitalizations. Therefore, the primary analysis retained the outcome and factors in crude, unstandardized form and included demographic covariates to account for differences in state population structure. These variables were derived from the U.S. Census’ 2022 American Community Survey [11] and included: (i) the proportion of adults aged ≥ 65 years, (ii) the proportion of male adults aged ≥ 65 years, and (iii) the proportion of female adults aged < 65 years. Together, these variables reflect both the independent distributions of age and sex as well as conditional age–sex subgroups.

### Statistical analysis

We computed descriptive statistics for Long COVID prevalence and all factors, including SARS-CoV-2 incidence per person-year, COVID-19 hospitalizations per person-year, COVID-19 vaccine coverage, and multimorbidity. We then conducted univariable linear regressions to assess the association between Long COVID prevalence and each factor individually. Finally, we fit a multivariable model that incorporated all four state-level factors along with the three age and sex variables defined above.

Regression coefficients, β, and corresponding 95% CI were reported. Coefficients were scaled to improve interpretability. Specifically, β represents the average difference in state-level Long COVID prevalence, in percentage points, associated with the specified increment in each variable: per 10 reported cases per 1,000 person-years for SARS-CoV-2 incidence, per 1 COVID-19 hospitalization per 1,000 person-years for COVID-19 hospitalizations, and per 10 percentage-point increase for COVID-19 vaccine coverage, multimorbidity.

State-level Long COVID prevalence was derived from BRFSS survey estimates with known standard errors, and standard errors were also estimated for COVID-19 vaccine coverage. We therefore propagated sampling variability in the outcome (i.e., Long COVID prevalence) and one factor, COVID-19 vaccine coverage, into the model. Specifically, we generated 1,000 Monte Carlo samples of state-level Long COVID prevalence and COVID-19 vaccine coverage independently from normal distributions centered at their mean estimates, with dispersion defined by their corresponding standard errors. Because variance estimates were not available for the other factors, including SARS-CoV-2 incidence, COVID-19 hospitalizations and multimorbidity, these factors were held fixed. Each simulation produced a complete analytic data set, to which we fit the full multivariable model. Regression coefficients and variance-covariance matrices were retained and pooled using Rubin’s rules [15], producing pooled estimates and total variances that incorporated both within- and between-simulation variability.

We assessed model diagnostics using correlation matrix and variance inflation factors (VIFs). We also conducted the following sensitivity analyses: 1) leave-one-state-out models; and 2) a shortened reporting window for SARS-CoV-2 incidence, ending April 1, 2023, rather than May 10, 2023, to account for early cessation of reporting in some states and intermittent missing reporting.

Long COVID prevalence was estimated from the BRFSS survey, using SAS® software, version 9.4 (SAS Institute Inc., Cary, NC). Statistical modeling and simulation-based inference were conducted in R version 4.4.0 (R Foundation for Statistical Computing, Vienna, Austria). Statistical significance was defined as p < 0.05, with results at p < 0.01 and p < 0.001 noted as stronger levels of significance. Analytic code is available in https://github.com/CDCgov/Ecological-analysis-of-Long-COVID/releases/tag/v1.0.

### Ethical considerations

This study used de-identified, publicly available secondary data. This activity was reviewed by CDC, deemed not research, and conducted consistent with applicable federal law and CDC policy (45 C.F.R. part 46.102(l) [(2)], 21 C.F.R. part 56; 42 U.S.C. Sect. 241(d); 5 U.S.C. Sect. 552a; 44 U.S.C. Sect. 3501 et seq.).

## Results

### Descriptive statistics

During the analytic window of this analysis (October 1, 2022 through September 30, 2023; Fig 1), adult Long COVID prevalence displayed interstate heterogeneity, ranging from 4.7% in Maryland to 9.7% in West Virginia, with a median of 6.6% (Q1–Q3 5.9%–7.3%) (Fig 2). State-level factors likewise varied across the 48 states (Table 1). SARS-CoV-2 case incidence per person-year averaged 0.05 (SD = 0.01), with a median of 0.05 (Q1–Q3: 0.043–0.058) and a range of 0.034–0.076. COVID-19 hospitalizations per person-year were lower in magnitude but similarly heterogeneous, averaging 0.003 (SD = 0.001), with an interquartile range tightly centered around 0.003 and ranged from 0.002 to 0.004. COVID-19 vaccine coverage was more variable, averaging 28.5% (SD = 6.9%), with a median of 27.5% (Q1–Q3: 22.7%–33.3%) and a range of 15.8%–45.9%. Finally, prevalence of multimorbidity (≥3 chronic conditions) averaged 18.6% (SD = 3.9%), with a median of 18.8% (Q1–Q3: 15.8%–21.3%) and a range of 10.8%–29.0%.

**Table 1:** Descriptive Statistics of Long COVID Prevalence and State-Level Factors.

	Mean (SD)	Median (Q1-Q3)	Range
Long COVID prevalence (%)	6.7 (1.1)	6.6 [5.9 – 7.3]	4.7–9.7
SARS-CoV-2 incidence per person-year	0.05 (0.01)	0.05 [0.043 – 0.058]	0.034–0.076
COVID-19 hospitalizations per person-year	0.003 (0.001)	0.003 [0.003 – 0.003]	0.002–0.004
COVID-19 vaccine coverage (%)	28.5 (6.9)	27.5 [22.7 – 33.3]	15.8–45.9
Prevalence of ≥ 3 chronic conditions (%)	18.6 (3.9)	18.8 [15.8 – 21.3]	10.8–29

Data were obtained from CDC COVID-19 Response Data (SARS-CoV-2 incidence) [10], NHSN (COVID-19 hospitalization rates) [12], NIS-ACM (COVID-19 vaccine coverage) [13], and CDC CFA (Prevalence ≥3 chronic conditions). Abbreviations: BRFSS, Behavioral Risk Factor Surveillance System; NHSN, National Healthcare Safety Network; NIS-ACM, NIS–Adult COVID Module; CDC, Centers for Disease Control and Prevention; CI, confidence interval; CFA, Center for Forecasting and Outbreak Analytics.

**Fig 1:**
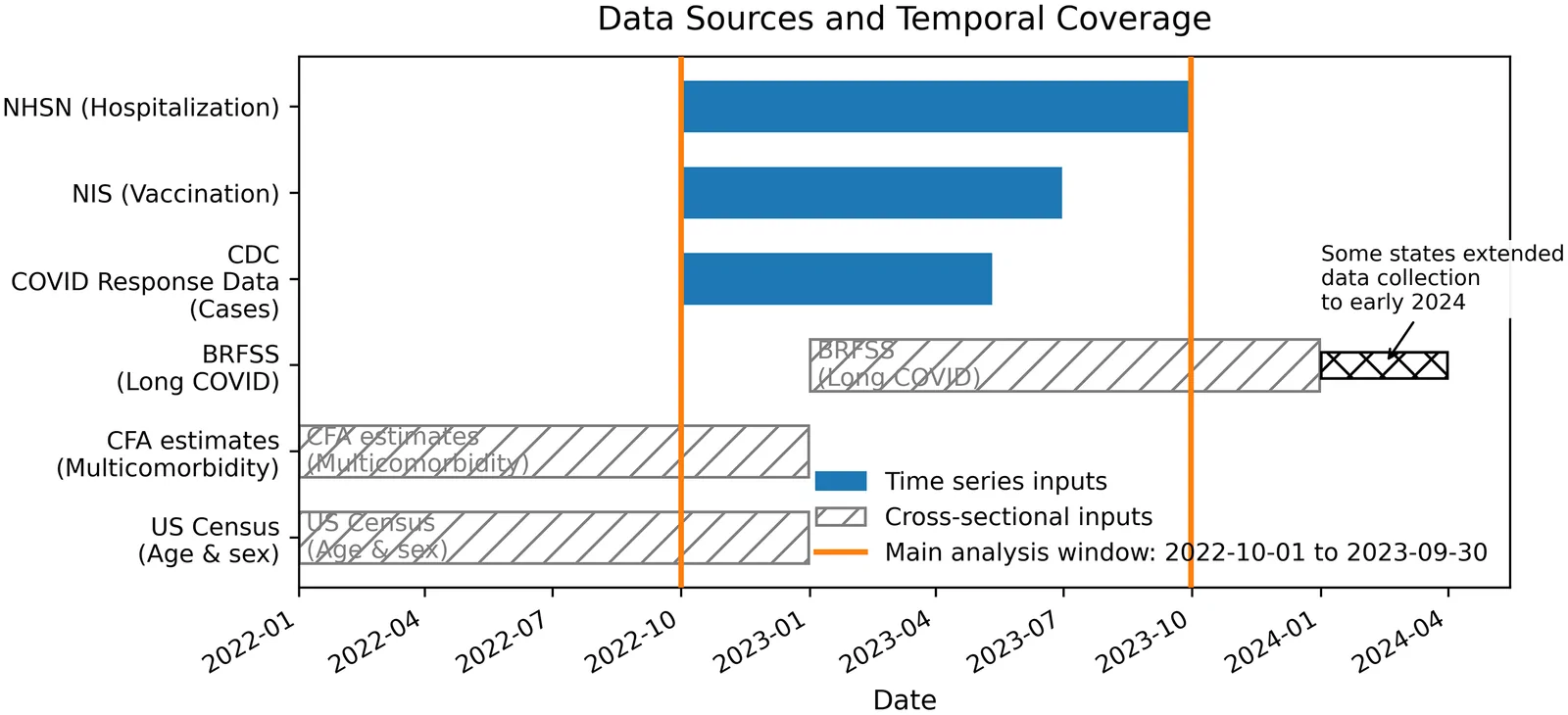
Temporal Coverage of Data Sources and Main Analysis Window. Solid bars represent time series inputs (COVID-19 hospitalizations, COVID-19 vaccine coverage, SARS-CoV-2 incidence), while hatched bars indicate cross-sectional data sources (e.g., demographics, multimorbidity, Long COVID estimates). The orange vertical lines denote the start and end of the main analysis period (October 2022 to October 2023).

**Fig 2:**
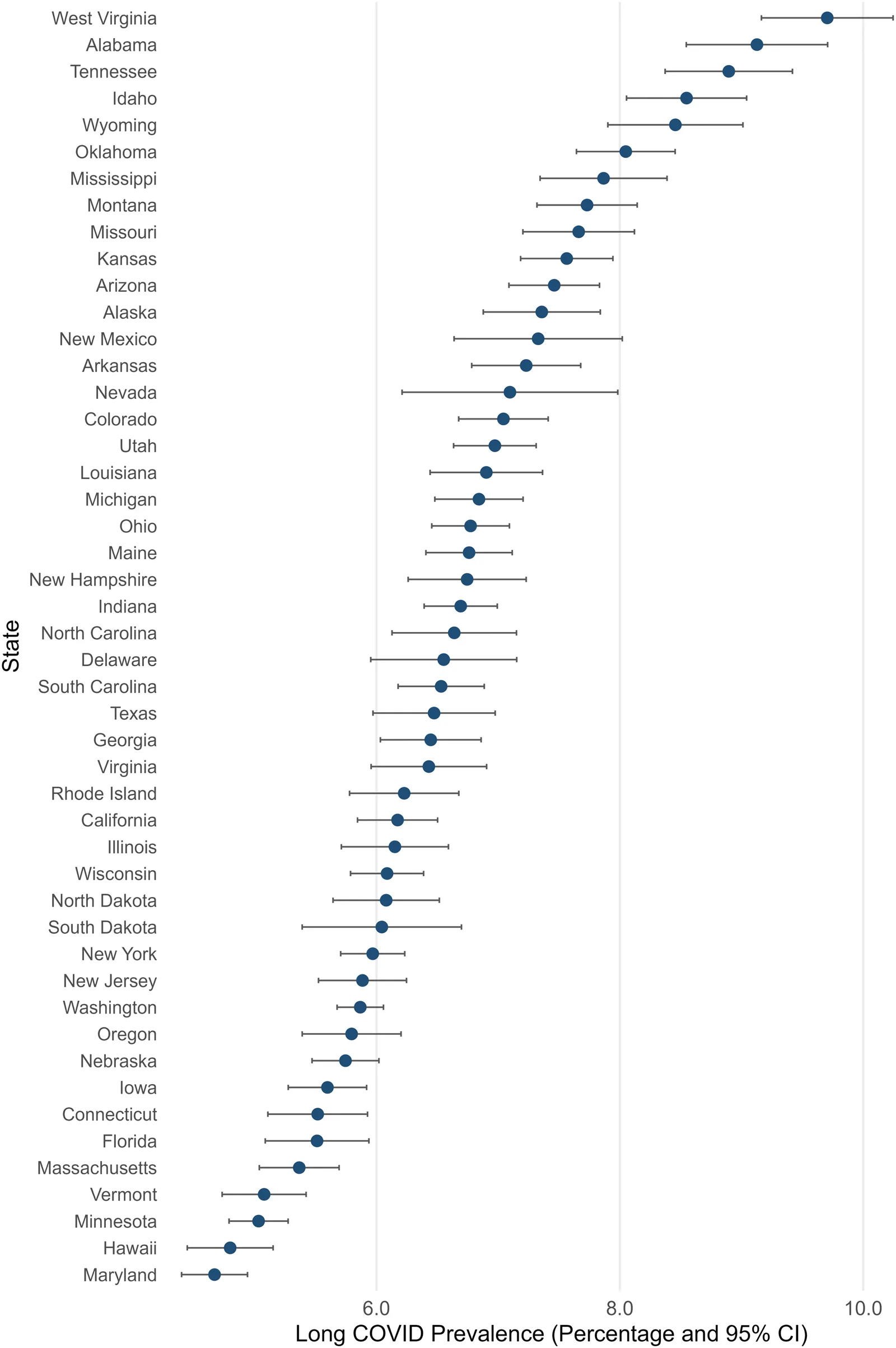
Prevalence of Long COVID among Adults by State with 95% CI. Estimated prevalence of Long COVID among U.S. adults by state in the 2023 BRFSS [9], ordered from highest to lowest prevalence. Error bars represent 95% CI of the estimates derived from the 2023 BRFSS data. Kentucky and Pennsylvania were not included in the public-use 2023 BRFSS data set. Non-state jurisdictions were also excluded from analysis. Abbreviations: BRFSS, Behavioral Risk Factor Surveillance System; CI, confidence interval.

### Univariable regression

In univariable regressions (Table 2), state-level vaccine coverage and multimorbidity were significantly associated with Long COVID prevalence. A 10 percentage-point higher COVID-19 vaccine coverage was associated with a 0.97 percentage-point lower Long COVID prevalence (p < 0.001). Similarly, states with a higher proportion of adults with multimorbidity had higher Long COVID prevalence (β = 1.30; p = 0.001). SARS-CoV-2 incidence and COVID-19 hospitalization were not significantly associated with Long COVID prevalence (both p > 0.05).

**Table 2:** Associations of State-Level Factors with Adult Long COVID Prevalence Across Univariable, Multivariable, and Sampling–Error–Adjusted Models of Long COVID Prevalence.

Model	Univariable Regression		Multivariable Regression		Multivariable Regression (Uncertainty analysis)	
State-Level Factor	β (95% CI)	p-value	β (95% CI)	p-value	β (95% CI)	p-value
SARS-CoV-2 incidence per person-year	0.15 (−0.20, 0.49)	0.39	0.35 (0.06, 0.64)	0.02*	0.35 (−0.005, 0.71)	0.053
COVID-19 hospitalizations per person-year	−0.37 (−0.89, 0.16)	0.17	−0.08 (−0.52, 0.37)	0.72	−0.04 (−0.59, 0.51)	0.78
COVID-19 vaccine coverage	−0.97 (−1.35, −0.58)	<0.001	−0.74 (−1.18, −0.30)	<0.001	−0.72 (−1.26, −0.17)	0.01**
Prevalence ≥3 chronic conditions	1.30 (0.54, 2.06)	0.001**	0.68 (−0.16, 1.53)	0.11	0.70 (−0.35, 1.74)	0.19

Results from univariable, multivariable model, and uncertainty analyses estimating associations between state-level factors and adult Long COVID prevalence (2023 BRFSS) [9], incorporating survey error from BRFSS estimates and NIS-ACM estimates. Data for examined factors were obtained from CDC COVID-19 Response Data (SARS-CoV-2 incidence) [10], NHSN (COVID-19 hospitalization rates) [12], NIS-ACM (COVID-19 vaccine coverage) [13], and CDC CFA (Prevalence ≥3 chronic conditions). *p < 0.05, **p < 0.01. β represents the scaled regression coefficients, interpreted as the average magnitude of change in Long COVID prevalence with the specified increments in a given factor (a positive β means an increase in Long COVID prevalence and a negative β means a decrease). Specifically, per 10 reported cases per 1,000 person-years for SARS-CoV-2 incidence, per 1 COVID-19 hospitalization per 1,000 person-years for COVID-19 hospitalizations, and per 10 percentage-point increase for COVID-19 vaccine coverage, multimorbidity, and demographic variables. CI represents confidence interval of regression coefficient β. Abbreviations: BRFSS, Behavioral Risk Factor Surveillance System; CDC, Centers for Disease Control and Prevention; CFA, Center for Forecasting and Outbreak Analytics; CI, confidence interval; NHSN, National Healthcare Safety Network; NIS-ACM, NIS–Adult COVID Module.

Fig 3 presents the scatterplot of state-level COVID-19 vaccine coverage and Long COVID prevalence, with a fitted regression line and 95% CIs. The plot is consistent with a negative state-level association between COVID-19 vaccine coverage and Long COVID prevalence. Additional scatterplots of Long COVID prevalence against each state-level variable are provided in S1 Text Fig A–F.

**Fig 3:**
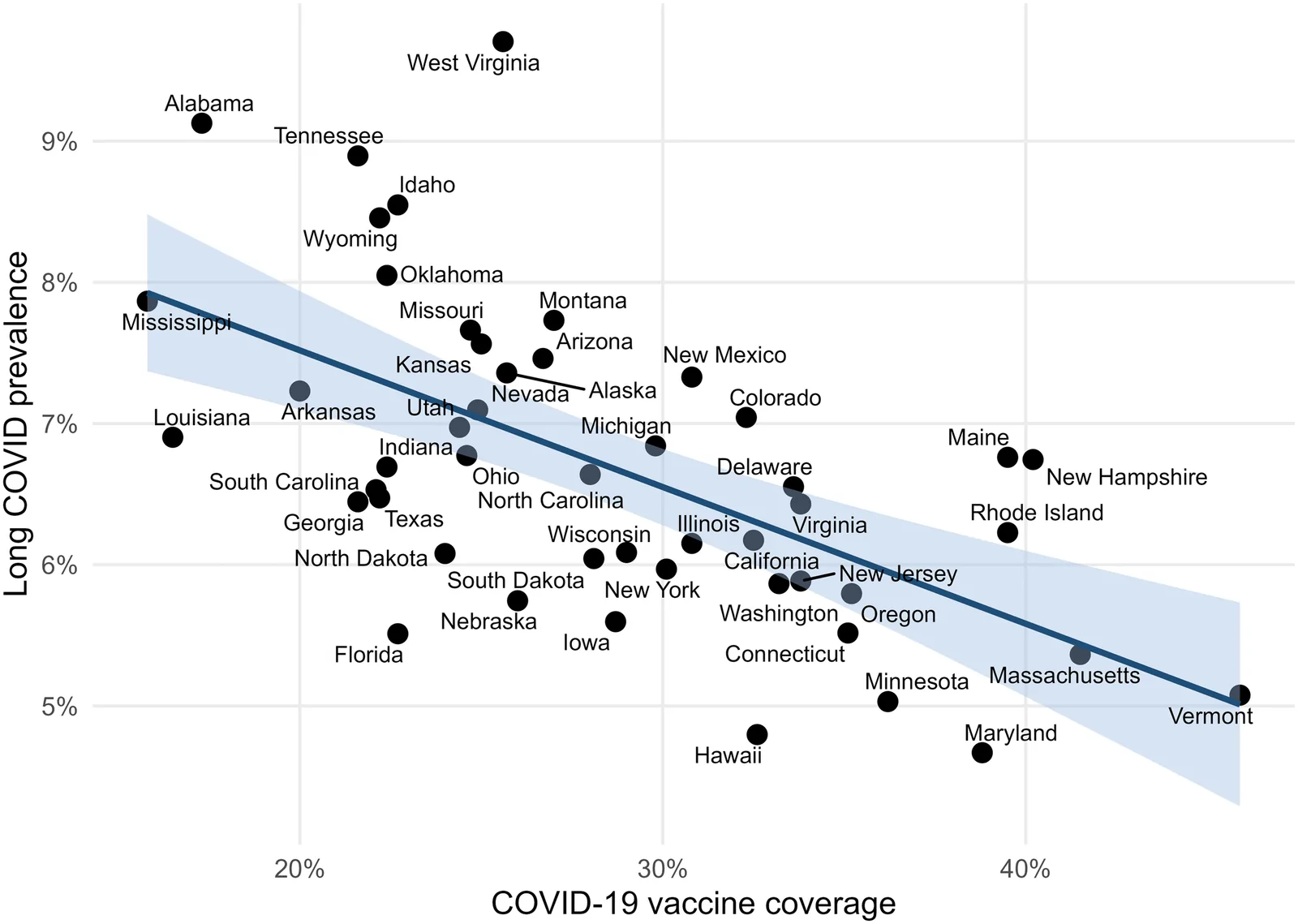
Scatterplot of Adult Long COVID Prevalence by Cumulative COVID-19 Vaccine Coverage by State, 2023. Long COVID data are from the 2023 BRFSS [9]. Kentucky and Pennsylvania were not included in the BRFSS public-use dataset; vaccine coverage data are from the NIS-ACM [13]. Abbreviations: BRFSS, Behavioral Risk Factor Surveillance System; NIS-ACM, NIS–Adult COVID Module.

### Multivariable regression

In the multivariable model including all four state-level factors and adjusted for age and sex distribution (proportion adults ≥ 65 years old; proportion male ≥ 65 years; proportion female < 65 years), vaccine coverage remained negatively associated with Long COVID prevalence (β = –0.74, p < 0.001) (Table 2). SARS-CoV-2 incidence was positively associated with Long COVID prevalence after adjustment (β = 0.35; p = 0.02). The association between multimorbidity and Long COVID prevalence was reduced in magnitude after adjustment and was no longer statistically significant (β = 0.68, p = 0.11). COVID-19-hospitalization rates were not statistically associated with Long COVID prevalence in the fully adjusted model. The adjusted R^2^ was 0.50.

The correlation matrix between factors considered in our model is presented in Fig G in S1 Text. The Spearman correlation between the proportion of males among adults aged ≥65 years and the proportion of females among adults a ged <65 years was −0.79. The Spearman correlation between multimorbidity and COVID-19 vaccine coverage was −0.52. All VIFs were <5 (S1 Text, Table D), suggesting that multicollinearity was not severe in the fully adjusted model.

### Uncertainty analysis

The results using the Monte Carlo simulations were similar to those from the multivariable regression using point estimates of Long COVID prevalence without standard error incorporated in the outcome (Table 2); state-level COVID-19 vaccine coverage remained significantly negatively associated with Long COVID prevalence (mean β = −0.72; p < 0.01) while SARS-CoV-2 incidence fell slightly short of statistical significance (mean β = 0.35; p = 0.053). The other state-level factors, multimorbidity and hospitalizations were not associated with Long COVID prevalence.

### Sensitivity analysis

Sensitivity analyses using a shortened reporting window for SARS-CoV-2 incidence (S1 Text, Table E), and leave-one-state-out models (S1 Text, Table F) were generally consistent with the primary analysis. Across these analyses, higher state-level COVID-19 vaccine coverage remained negatively associated with Long COVID prevalence.

## Discussion

There are two major findings in this ecological study. First, in addition to Long COVID prevalence, all state-level factors we examined, SARS-CoV-2 incidence, COVID-19 hospitalizations, COVID-19 vaccine coverage, and multimorbidity, showed marked interstate variation. Second, when these factors were examined together in a multivariable model, vaccine coverage was the only factor consistently and significantly associated with Long COVID prevalence. Higher state-level COVID-19 vaccine coverage was associated with a lower Long COVID prevalence. SARS-CoV-2 incidence showed a modest positive association with Long COVID prevalence in the adjusted model, although this association was not robust after accounting for uncertainty or when using a shorter time window for SARS-CoV-2 incidence. These findings were robust to uncertainty analyses which accounted for margin of error in state-level Long COVID and vaccine coverage estimates. Our findings underscore the potential importance of COVID-19 vaccination and SARS-CoV-2 infection prevention in mitigating adult Long COVID at the state-level. These results may help state and local health departments identify populations with higher risk and support development of targeted prevention strategies.

A growing body of cohort studies and meta-analyses demonstrates that recent COVID-19 vaccination lowers the probability of developing Long COVID [5,14,16,17]. Our findings were consistent with the body of evidence. The negative correlation between state-level COVID-19 vaccine coverage and Long COVID prevalence persisted after controlling for all other factors. Some studies have reported conflicting findings [18,19], which may be due to differences in Long COVID definitions, follow-up intervals, or dominant variant.

Individuals with multiple chronic conditions are more likely to have severe outcomes after SARS-CoV-2 infection [20]. In our study, multimorbidity was positively associated with Long COVID prevalence in the univariable model but not after adjustment for all other state-level factors and age and sex variables. This attenuation may indicate that multimorbidity overlaps with other measured state-level characteristics included in the model.

At the individual-level, severe acute COVID-19 illness, including hospitalization, has been shown to increase the risk of developing Long COVID [4,21]. However, our study did not find a significant association between COVID-19 hospitalization and Long COVID prevalence, either before or after adjustment. This difference may reflect the ecological nature of our analysis, as associations observed at the individual level do not necessarily correspond to population-level patterns or the differences in the study populations (BRFSS 2023) and other reports.

To our knowledge, this study is the first to systematically investigate associations between state-level COVID-19 metrics and adult Long COVID prevalence. We used the best available data sources and aligned the timeframes of these factors with BRFSS definition of Long COVID (i.e., symptoms lasting 3 or more months following COVID-19). In addition, this study accounted for uncertainty in the outcome by incorporating the margin of error for Long COVID and COVID-19 vaccine coverage estimates, although uncertainty in the other variables could not be incorporated.

The findings should be interpreted in the context of the study design, data sources, and data analyses. First, as an ecological study, we cannot infer causal or individual-level relationships between state-level factors and Long COVID prevalence. Second, temporal alignment between the state-level factors and Long COVID remains a limitation. All examined factors except multimorbidity were measured as weekly or monthly aggregates, whereas the 2023 BRFSS Long COVID measure reflects cross-sectional reports of symptoms persisting for three or more months following COVID-19. Although we defined the three-month period preceding Long COVID to capture lags, this window may not capture the relevant exposure history for all prevalent Long COVID cases or the precise temporal lag at the individual level. Some Long COVID cases reported in 2023 may have resulted from infections occurring in prior years. Monthly or quarterly BRFSS estimates could not be used due to insufficient sample sizes. This temporal misalignment is particularly relevant to COVID-19 vaccine coverage, which may not reflect vaccination status at the time of the infection preceding Long COVID rather than a broader indicator of state-level characteristics such as healthcare access, health behavior, socioeconomic status, or other factors. Further, because BRFSS did not capture information about Long COVID symptom duration, we were unable to determine if state-level Long COVID burden was driven by newly acquired or ongoing cases (e.g., Long COVID acquired in 2020). Third, SARS-CoV-2 incidence and COVID-19 hospitalization rates are subject to under-ascertainment and variation in testing, reporting practices, and access to testing [22]. As a result, these measures may not fully capture the true burden of infection or severe illness. Fourth, Long COVID may also be under-ascertained in BRFSS, particularly among individuals with asymptomatic or mild SARS-CoV-2 infection who may be less likely to test for COVID-19 and therefore less likely to be identified through the survey’s Long COVID questions. Conversely, the absence of a lab-confirmed infection requirement could potentially lead to overestimation of Long COVID, as Long COVID symptoms (e.g., fatigue, cough) are not unique to SARS-CoV-2 infection [23]. Finally, BRFSS does not collect information on Long COVID phenotype, reinfection history, timing of vaccination relative to COVID-19 illness, or access to care, all of which may influence Long COVID prevalence estimates and observed state-level associations.

Higher state-level COVID-19 vaccine coverage was consistently associated with lower adult Long COVID prevalence. Together with existing evidence [24,25], these ecological findings support further investigation of vaccination and other state-level determinants of Long COVID but should not be interpreted as evidence of an individual-level or causal effect. Future surveillance and research may benefit from capturing Long COVID across acute illness severity, Long COVID phenotype, SARS-CoV-2 testing history, COVID-19 vaccination and reinfection status, and access to care.

## Supporting information

S1 Text Supplemental materials. (DOCX)
