# Supplementary material for: Long COVID prevalence among U.S. adults: A state-level ecological analysis of associations with SARS-CoV-2 incidence, COVID-19 hospitalization rates, COVID-19 vaccine coverage, and multimorbidity
Source: PLoS One. 2026 Nov 3;21(11):e0345433. doi: 10.1371/journal.pone.0345433 (PMC13730352; doi:10.1371/journal.pone.0345433)
Supplement: Supplementary file 1 [file pone.0345433.s001.docx]

**Table A. Data Source Descriptions**

| **Variable Name** | **Definition** | **Data Source** | **Time Frame** | **Cadence** | **Calculation Notes** | **Uncertainty Estimated** |
| --- | --- | --- | --- | --- | --- | --- |
| SARS-CoV-2 incidence per person-years | Total confirmed or probable COVID-19 cases per person-years | Centers for Disease Control and Prevention. Weekly United States COVID-19 cases and deaths by state - ARCHIVED. June 1, 2023. Available at: <https://data.cdc.gov/Case-Surveillance/Weekly-United-States-COVID-19-Cases-and-Deaths-by-/pwn4-m3yp>. | Oct 1, 2022 – May 10, 2023 | Weekly | (Total reported cases across observed weeks / state population / number of observed weeks) x 52 | No |
| COVID-19 hospitalization per person-years | Total adult COVID-19 hospitalizations per person-years | Centers for Disease Control and Prevention. Weekly Hospital Respiratory Data (HRD) Metrics by Jurisdiction, National Healthcare Safety Network (NHSN) Available at:<https://healthdata.gov/CDC/Weekly-Hospital-Respiratory-Data-HRD-Metrics-by-Ju/n3kj-exp9/about_data> | Oct 1, 2022 – Sep 30, 2023 | Weekly | (Sum of weekly COVID-19 admission rates per 100,000 population / number of observed weeks / 100,000) x 52 | No |
| COVID-19 vaccine coverage | Proportion of adults who received a bivalent booster | National Immunization Survey Adult COVID Module (NIS-ACM): Trends in Vaccination Status and Intent Additional information in:<https://data.cdc.gov/Vaccinations/National-Immunization-Survey-Adult-COVID-Module-NI/akkj-j5ru/about_data> | Oct 1, 2022 – Jun 30, 2023 | Monthly | Survey weights and complex survey design adjustments using March-June data | Yes |
| Multimorbidity | Proportion of adults with ≥3 chronic health conditions (multimorbidity) | Centers for Disease Control and Prevention. CFA model estimates using BRFSS, NHIS, claims, and consumer data | Modeled using 2022–2023 data | Cross-sectional | MRP-predicted cases Predicted counts of adults with three or more conditions) ÷ population estimates) | No |
| Long COVID prevalence | State-level prevalence of current Long COVID among adults | 2023 BRFSS <https://www.cdc.gov/brfss/index.html> | Jan – Dec 2023 | Cross-sectional | Based on self-report of prior COVID-19 infection and symptoms lasting ≥3 months that were not present prior to having COVID-19; estimates weighted using BRFSS final sampling weights | Yes |

Abbreviations: BRFSS, Behavioral Risk Factor Surveillance System; CFA, Center for Forecasting and Outbreak Analytics; NHIS, National Health Interview Survey.

**Table B. Prevalence of Multimorbidity by State**

| **State** | **Prevalence** |
| --- | --- |
| Alabama | 23.0% |
| Alaska | 15.8% |
| Arizona | 17.6% |
| Arkansas | 23.7% |
| California | 19.9% |
| Colorado | 16.1% |
| Connecticut | 16.5% |
| Delaware | 21.3% |
| Florida | 21.3% |
| Georgia | 21.8% |
| Hawaii | 13.6% |
| Idaho | 18.2% |
| Illinois | 18.0% |
| Indiana | 19.6% |
| Iowa | 21.4% |
| Kansas | 20.0% |
| Louisiana | 29.0% |
| Maine | 18.4% |
| Maryland | 14.1% |
| Massachusetts | 12.3% |
| Michigan | 21.0% |
| Minnesota | 14.0% |
| Mississippi | 21.8% |
| Missouri | 19.4% |
| Montana | 17.1% |
| Nebraska | 22.6% |
| Nevada | 20.6% |
| New Hampshire | 14.2% |
| New Jersey | 16.1% |
| New Mexico | 18.3% |
| New York | 15.8% |
| North Carolina | 18.8% |
| North Dakota | 10.8% |
| Ohio | 22.9% |
| Oklahoma | 21.7% |
| Oregon | 14.3% |
| Rhode Island | 22.3% |
| South Carolina | 19.4% |
| South Dakota | 16.4% |
| Tennessee | 23.9% |
| Texas | 18.9% |
| Utah | 11.6% |
| Vermont | 15.8% |
| Virginia | 19.6% |
| Washington | 13.4% |
| West Virginia | 26.8% |
| Wisconsin | 20.8% |
| Wyoming | 14.7% |

Data were from unpublished 2024 estimates produced by the CDC’s Center for Forecasting and Outbreak Analytics (CFA). Multimorbidity here represents the prevalence of >= 3 chronic conditions that are at increased risk of COVID-19 hospitalization, including asthma, obesity, diabetes, heart condition, current or former smoking, tuberculosis, cerebrovascular disease, chronic liver disease, chronic kidney disease, chronic lung disease, mental health disorder, cystic fibrosis, HIV, immunodeficiency, transplantation, immunosuppression, cancer, pregnancy, disability and neurocognitive or dementia-related conditions.

**Table C.  COVID-19 Vaccine Coverage and its standard error by State**

| **State** | **Prevalence** | **Standard Error** |
| --- | --- | --- |
| Alabama | 17.3% | 0.7% |
| Alaska | 25.7% | 0.8% |
| Arizona | 26.7% | 0.8% |
| Arkansas | 20.0% | 0.7% |
| California | 32.5% | 0.9% |
| Colorado | 32.3% | 0.9% |
| Connecticut | 35.1% | 2.1% |
| Delaware | 33.6% | 0.9% |
| Florida | 22.7% | 1.0% |
| Georgia | 21.6% | 1.4% |
| Hawaii | 32.6% | 1.0% |
| Idaho | 22.7% | 1.0% |
| Illinois | 30.8% | 0.6% |
| Indiana | 22.4% | 0.8% |
| Iowa | 28.7% | 1.1% |
| Kansas | 25.0% | 0.8% |
| Louisiana | 16.5% | 0.6% |
| Maine | 39.5% | 1.0% |
| Maryland | 38.8% | 1.2% |
| Massachusetts | 41.5% | 0.9% |
| Michigan | 29.8% | 0.9% |
| Minnesota | 36.2% | 0.9% |
| Mississippi | 15.8% | 0.7% |
| Missouri | 24.7% | 0.9% |
| Montana | 27.0% | 0.8% |
| Nebraska | 26.0% | 1.0% |
| Nevada | 24.9% | 0.8% |
| New Hampshire | 40.2% | 1.0% |
| New Jersey | 33.8% | 1.0% |
| New Mexico | 30.8% | 0.8% |
| New York | 30.1% | 0.8% |
| North Carolina | 28.0% | 0.9% |
| North Dakota | 24.0% | 0.9% |
| Ohio | 24.6% | 1.1% |
| Oklahoma | 22.4% | 0.9% |
| Oregon | 35.2% | 0.8% |
| Rhode Island | 39.5% | 1.3% |
| South Carolina | 22.1% | 0.8% |
| South Dakota | 28.1% | 0.8% |
| Tennessee | 21.6% | 1.1% |
| Texas | 22.2% | 0.7% |
| Utah | 24.4% | 1.4% |
| Vermont | 45.9% | 1.5% |
| Virginia | 33.8% | 0.8% |
| Washington | 33.2% | 1.4% |
| West Virginia | 25.6% | 0.8% |
| Wisconsin | 29.0% | 0.8% |
| Wyoming | 22.2% | 0.9% |

**Data estimates were from** NIS-ACM, NIS–Adult COVID Module (1)

**Table D.  Variance Inflation Factors for Factors in the Multivariable Regression Model**

| **Factor** | **VIF** |
| --- | --- |
| SARS-CoV-2 case incidence per person-year | 1.40 |
| COVID-19 hospitalizations per person-year | 1.39 |
| COVID-19 vaccine coverage | 1.70 |
| Prevalence of multimorbidity (>=3 chronic conditions) | 1.99 |
| Proportion of adults >=65 years | 1.65 |
| Proportion of male among adults >=65 years | 4.71 |
| Proportion of female among adults <65 years | 3.81 |

VIF = variance inflation factor. VIFs were calculated from multivariable models including all listed factors. Larger VIF values indicate greater multicollinearity among factors.
**Table E. Leave-One-State-Out Sensitivity Analysis of the Multivariable Regression Model**

Each analysis refits the multivariable model after excluding one state at a time. The full-data beta is the coefficient estimated using all states. Minimum and maximum leave-one-out beta values show the range of coefficients across state-exclusion models. Max absolute percent change is the largest absolute percent difference from the full-data beta. The state with max change is the excluded state that produced the largest coefficient change. P < 0.05 in all analyses indicates whether the predictor remained statistically significant in every leave-one-state-out model.

**Table F. Sensitivity Analysis Using Shorter Reporting Windows for SARS-CoV-2 Incidence.**

| **Factor** | **Reported increment** | **Beta, percentage-point change** | **95% CI** | **P value** |
| --- | --- | --- | --- | --- |
| SARS-CoV-2 case incidence per person-year | per 10 reported cases/ 1,000 persons-year | 0.15 | -0.08, 0.38 | 0.19 |
| COVID-19 hospitalizations per person-year | per 1 hospitalization/ 1,000 person-year | -0.17 | -0.63, 0.30 | 0.47 |
| COVID-19 vaccine coverage | per 10 percentage-point increase | -0.76 | -1.23, -0.30 | <0.001 |
| Prevalence of multimorbidity (>=3 chronic conditions) | per 10 percentage-point increase | 0.82 | -0.06, 1.70 | 0.07 |
| Proportion of adults >=65 years | per 10 percentage-point increase | 0.07 | -1.39, 1.52 | 0.93 |
| Proportion of male among adults >=65 years | per 10 percentage-point increase | 4.44 | 0.86, 8.02 | 0.02 |
| Proportion of female among adults <65 years | per 10 percentage-point increase | 3.48 | -1.35, 8.30 | 0.15 |

Beta estimates represent percentage-point changes in crude Long COVID prevalence for the reported increment of each factor. This sensitivity analysis used a shorter SARS-CoV-2 incidence window ending April 1, compared with May 10, 2023 in the main analysis. The model included all listed factors. CI = confidence interval. P values are from the multivariable regression model.

**Table G. Multivariable regression diagnostic statistics and identification of potentially influential observations by state.**

| **State** | **Leverage** | **Cook's distance** |
| --- | --- | --- |
| Alabama | 0.14 | 0.06 |
| Alaska | 0.42 | 0.02 |
| Arizona | 0.06 | 0.00 |
| Arkansas | 0.12 | 0.00 |
| California | 0.14 | 0.00 |
| Colorado | 0.11 | 0.03 |
| Connecticut | 0.21 | 0.00 |
| Delaware | 0.16 | 0.01 |
| Florida | 0.20 | 0.15 |
| Georgia | 0.24 | 0.00 |
| Hawaii | 0.28 | 0.02 |
| Idaho | 0.15 | 0.03 |
| Illinois | 0.09 | 0.00 |
| Indiana | 0.08 | 0.00 |
| Iowa | 0.18 | 0.03 |
| Kansas | 0.07 | 0.01 |
| Louisiana | 0.21 | 0.11 |
| Maine | 0.19 | 0.01 |
| Maryland | 0.20 | 0.01 |
| Massachusetts | 0.26 | 0.01 |
| Michigan | 0.04 | 0.00 |
| Minnesota | 0.07 | 0.01 |
| Mississippi | 0.22 | 0.00 |
| Missouri | 0.05 | 0.01 |
| Montana | 0.22 | 0.00 |
| Nebraska | 0.10 | 0.03 |
| Nevada | 0.15 | 0.00 |
| New Hampshire | 0.09 | 0.02 |
| New Jersey | 0.26 | 0.01 |
| New Mexico | 0.12 | 0.00 |
| New York | 0.27 | 0.09 |
| North Carolina | 0.10 | 0.00 |
| North Dakota | 0.39 | 0.03 |
| Ohio | 0.07 | 0.00 |
| Oklahoma | 0.10 | 0.01 |
| Oregon | 0.11 | 0.00 |
| Rhode Island | 0.23 | 0.00 |
| South Carolina | 0.18 | 0.08 |
| South Dakota | 0.09 | 0.01 |
| Tennessee | 0.12 | 0.03 |
| Texas | 0.14 | 0.00 |
| Utah | 0.32 | 0.00 |
| Vermont | 0.27 | 0.02 |
| Virginia | 0.09 | 0.00 |
| Washington | 0.17 | 0.00 |
| West Virginia | 0.24 | 0.34 |
| Wisconsin | 0.08 | 0.03 |
| Wyoming | 0.20 | 0.03 |

Cook's distance measures how much a specific data point affects the results of a regression model. Leverage measures how far that observation’s predictor values are from the overall mean of the predictors.

**Figure A. Association Between Multimorbidity and Long COVID Prevalence among Adults by State.** State-level estimates of Long COVID prevalence among U.S. adults (2) plotted against state-level prevalence of multimorbidity (prevalence of >=3 chronic conditions) (See Table S2). Each point represents a state, and state labels identify individual estimates. The solid line represents the fitted linear trend, and the shaded area represents the 95% CI around the fitted trend. Kentucky and Pennsylvania were not included in the public-use 2023 BRFSS data set.

Abbreviations: BRFSS, Behavioral Risk Factor Surveillance System; CI, confidence interval.

**Figure B. Association Between COVID-19 Hospitalization and Long COVID Prevalence among Adults by State.** State-level estimates of Long COVID prevalence among U.S. adults (2) plotted against state-level cumulative COVID-19 hospitalization per 1,000 person-years (3). Each point represents a state, and state labels identify individual estimates. The solid line represents the fitted linear trend, and the shaded area represents the 95% CI around the fitted trend. Kentucky and Pennsylvania were not included in the public-use 2023 BRFSS data set. Note that we used COVID-19 hospitalization per person-year in the model, we presented it in per 1,000 person-years for a better visualization.

Abbreviations: BRFSS, Behavioral Risk Factor Surveillance System; CI, confidence interval.

**Figure C. Association Between SARS-CoV-2 Incidence and Long COVID Prevalence among Adults by State.** State-level estimates of Long COVID prevalence among U.S. adults (2) plotted against state-level cumulative SARS-CoV-2 incidence per 1,000 person-years (4). Each point represents a state, and state labels identify individual estimates. The solid line represents the fitted linear trend, and the shaded area represents the 95% CI around the fitted trend. Kentucky and Pennsylvania were not included in the public-use 2023 BRFSS data set. Note that we used SARS-CoV-2 incidence per person-year in the model, we presented it in per 1,000 person-years for better visualization.

Abbreviations: BRFSS, Behavioral Risk Factor Surveillance System; CI, confidence interval.

**Figure D. Association Between Proportion of Adults >=65 yrs and Long COVID Prevalence among Adults by State.** State-level estimates of Long COVID prevalence among U.S. adults (2) plotted against state-level proportion of adults >=65 yrs (5). Each point represents a state, and state labels identify individual estimates. The solid line represents the fitted linear trend, and the shaded area represents the 95% CI around the fitted trend. Kentucky and Pennsylvania were not included in the public-use 2023 BRFSS data set.

Abbreviations: BRFSS, Behavioral Risk Factor Surveillance System; CI, confidence interval.

**Figure E. Association Between Proportion of Females Among < 65 yrs and Long COVID Prevalence among Adults by State.** State-level estimates of Long COVID prevalence among U.S. adults (2) plotted against state-level proportion of females among <65 yrs (5). Each point represents a state, and state labels identify individual estimates. The solid line represents the fitted linear trend, and the shaded area represents the 95% CI around the fitted trend. Kentucky and Pennsylvania were not included in the public-use 2023 BRFSS data set.

Abbreviations: BRFSS, Behavioral Risk Factor Surveillance System; CI, confidence interval.

**Figure F. Association Between Proportion of Males Among >= 65 yrs and Long COVID Prevalence among Adults by State.** State-level estimates of Long COVID prevalence among U.S. adults (2) plotted against state-level proportion of males among >=65 yrs (5). Each point represents a state, and state labels identify individual estimates. The solid line represents the fitted linear trend, and the shaded area represents the 95% CI around the fitted trend. Kentucky and Pennsylvania were not included in the public-use 2023 BRFSS data set.

Abbreviations: BRFSS, Behavioral Risk Factor Surveillance System; CI, confidence interval.

**Figure G. Spearman Correlation Matrix of State-Level Factors Potentially Associated with Adult Long COVID Prevalence.** Pairwise Spearman correlations among state-level variables potentially associated with Long COVID prevalence. Color intensity reflects the strength and direction of correlation (blue: positive, red: negative).

**Figure H. Residuals versus fitted values for the multivariable model.** Residuals are plotted against fitted Long COVID prevalence, with the dashed horizontal line indicating zero residual.

**Figure I. Normal Q–Q plot of standardized residuals.** Standardized residuals are plotted against the corresponding theoretical quantiles from a standard normal distribution. The reference line indicates the expected relationship under approximate normality. The upper tails represent West Virginia.

**References**

1.	Centers for Disease Control and Prevention. National Immunization Survey Adult COVID Module (NIS-ACM): Trends in Vaccination Status and Intent (Version date: August 3, 2023) [Internet]. [cited 2024 Dec 10]. Available from: https://data.cdc.gov/Vaccinations/National-Immunization-Survey-Adult-COVID-Module-NI/akkj-j5ru/about_data

2.	Centers for Disease Control and Prevention. Atlanta, GA: U.S. Department of Health and Human Services, CDC [Internet]. 2024 [cited 2024 Sep 24]. Behavioral Risk Factor Surveillance System. Available from: https://www.cdc.gov/brfss/index.html

3.	Centers for Disease Control and Prevention, Division of Healthcare Quality Promotion, National Healthcare Safety Network (NHSN). Weekly United States Hospitalization Metrics by Jurisdiction, During Mandatory Reporting Period from August 1, 2020 to April 30, 2024, and for Data Reported Voluntarily Beginning May 1, 2024 (version date: February 23, 2025) [Internet]. [cited 2025 Mar 17]. Available from: https://data.cdc.gov/Public-Health-Surveillance/Weekly-United-States-Hospitalization-Metrics-by-Ju/aemt-mg7g/about_data

4.	Centers for Disease Control and Prevention. Weekly United States COVID-19 Cases and Deaths by State (version date: February 23, 2025) [Internet]. [cited 2025 Jun 10]. Available from: https://data.cdc.gov/Case-Surveillance/Weekly-United-States-COVID-19-Cases-and-Deaths-by-/pwn4-m3yp/about_data

5.	U.S. Census Bureau. Census.gov [Internet]. [cited 2025 Apr 16]. American Community Survey 5-Year Data (2022). Available from: https://www.census.gov/data/developers/data-sets/acs-5year.html
